# Late Enteral Feedings Are Associated with Intestinal Inflammation and Adverse Neonatal Outcomes

**DOI:** 10.1371/journal.pone.0132924

**Published:** 2015-07-14

**Authors:** Yelizaveta Konnikova, Munir M. Zaman, Meher Makda, Danila D’Onofrio, Steven D. Freedman, Camilia R. Martin

**Affiliations:** 1 Division of Newborn Medicine, Department of Pediatrics, Boston Children’s Hospital, Boston, Massachusetts, Unites States of America; 2 Division of Gastroenterology, Beth Israel Deaconess Medical Center, Boston, Massachusetts, United States of America; 3 Division of Translational Research, Beth Israel Deaconess Medical Center, Boston, Massachusetts, United States of America; 4 Department of Neonatology, Beth Israel Deaconess Medical Center, Boston, Massachusetts, United States of America; University of Florida, UNITED STATES

## Abstract

**Background:**

Morbidities of impaired immunity and dysregulated inflammation are common in preterm infants. Postnatal Intestinal development plays a critical role in the maturation of the immune system and is, in part, driven by exposure to an enteral diet.

**Objective:**

The aim of this study was to evaluate the influence of the timing of the first enteral feeding on intestinal inflammation and risk of disease.

**Methods:**

130 infants <33 weeks’ gestation were studied. Maternal and infant data were abstracted from the medical record. Single and multiplex ELISA assays quantified cytokines from fecal and serum samples at two weeks postnatal age.

**Results:**

A delay in enteral feedings after the third postnatal day is associated with a 4.5 (95% CI 1.8-11.5, p=0.002) fold increase in chronic lung disease, 2.9 (1.1-7.8, p=0.03) fold increase in retinopathy of prematurity, and 3.4 (1.2-9.8, p=0.02) fold increase in multiple comorbidities compared to infants fed on or before the third day. Additionally, a delay in the initiation of feedings is associated with increased fecal IL-8 levels and a decreased IL-10:IL-8 ratio.

**Conclusions:**

A delay in enteral feeding is associated with intestinal inflammation and increased risks of morbidities. To improve neonatal outcomes, early nutritional practices need to be reevaluated.

## Introduction

The intestine is the largest immune organ in the human body. Recent human and animal data suggest that postnatal gastrointestinal development as well as microbial colonization are critical for proper host immune function [1], [2]. This is supported by data in mice reared in germ free conditions where prevention of normal microbial colonization leads to underdeveloped gut associated lymphoid organs, decreased expression of MHC-II on antigen presenting cells and reduced IgA production [3], [4], [5]. In addition, germ free mice demonstrate impaired regulatory T-cells function [6]. Dysregulated and persistent intestinal inflammatory responses in critically ill patients have led to the emergent hypothesis in the adult literature that the gastrointestinal track is the “motor” of systemic illness [7].

Preterm infants must continue to undergo organ maturation in a suboptimal postnatal environment with superimposed exposures that increase the risk of organ injury and subsequent maldevelopment. A primary determinant of a number of morbidities associated with prematurity is the presence of inflammation, whether primary or secondary. The presence of inflammation during critical times of organ development can halt or alter normal developmental processes. As such, inflammation and infections early in life have been linked to chronic lung disease (CLD), retinopathy of prematurity (ROP) [8], necrotizing enterocolitis (NEC), periventricular leukomalacia (PVL) [9,10] and impaired neurodevelopment [11]. Similarly, chorioamnionitis and early neonatal sepsis have been linked with increased rates of CLD [12]. Numerous studies have shown that high levels of systemic cytokines such as IL-8 early in life are associated with increased risk for ROP [13] and increased IL-8, IL-6 and VEGF levels being associated with CLD [14]. Similarly, elevation of proinflammatory cytokines at 2 weeks of age have been shown to be associated with impaired mental and motor development at two years of age [11].

Due to the fear of severe feeding intolerance and NEC, preterm infants commonly experience a delay in initiating and advancing enteral feedings. However, there is compelling evidence from both animal studies as well as adult medicine that there might be drawbacks to such practices. Human and animal studies have shown that prolonged periods of oral fasting result in delayed mucosal growth, mucosal atrophy and dysregulated secretion of trophic hormones [15], [16], [17]. Delay in enteral nutrition both after trauma and post-surgeries increase mortality in adults [18], [19]. Furthermore, the lack of enteral feeding is associated with bacterial translocation, sepsis, and increased likelihood of systemic inflammatory response [17].

The intestinal tract undergoes rapid proliferation and growth during the third trimester or, in the case of the preterm infant, during the postnatal period while in the neonatal intensive care unit (NICU). Thus, early nutritional practices may have long lasting effects on the inflammatory state of the neonate and influence an infant’s risk of developing not only NEC but also other neonatal morbidities including CLD and ROP. Similar to the adult experience, the concept that inflammatory responses in the gut can drive or be the “motor” to systemic illness may be an inherent mechanism of neonatal disease pathogenesis.

We hypothesized that a delay in the initiation of enteral feedings would contribute to increased proinflammatory responses in the gut and predispose infants to neonatal morbidities. The aims of this study were (1) to evaluate the contribution of the timing of the first enteral feeding to the inflammatory state of the intestinal tract determined by quantification of inflammatory markers in fecal and serum samples, and (2) to assess if timing of the first enteral feeding was independently linked to neonatal morbidities.

## Materials and Methods

### Ethics Statement

The Beth Israel Deaconess Medical Center’s Institutional Review Board (IRB) approved the collection of discarded specimens for the clinical biorepository as well as the analyses conducted for this study (IRB protocol numbers: 2009P-000014 and 2009P-000193). Per our IRB approved protocol, verbal informed consent was obtained from the parents of infants enrolled in this study. All data were anonymized and de-identified for the study analysis.

### Cohort Selection

In this **retrospective** cohort study, infants were selected from a cohort of premature infants less than 33 weeks of gestation enrolled in the Infant Health Research Program at Beth Israel Deaconess Medical Center, Boston, an ongoing recruitment of premature infants with collection of discarded biological samples to evaluate the impact of nutrition on health and disease [20]. Serum and fecal samples were collected when available. No samples were obtained from enrolled infants specifically for the study.

For this study, infants were eligible for inclusion if fecal and serum samples were available at two weeks of age for analysis. Two weeks postnatal age was used as the time point of analysis as this represents the mean age to full enteral feedings and thus an optimal time point to evaluate the effect of enteral nutrition on infant outcomes. Given that infants with sepsis are likely to have systemic inflammation, those with culture positive sepsis were then excluded from the study. 130 infants met these inclusion criteria.

### Classification of Early versus Delayed Enteral Feedings

The objective of this study was to determine the effect, if any, of early versus late introduction of enteral feedings on intestinal and systemic health. To evaluate this objective, infants were categorized as either “Early” if they received their first enteral feed on or prior to the third day of life or “Late” if they received their first enteral feed after the third day of life. The cut-point at three days was used for two reasons. First, intestinal morphological changes are evident within 48–72 hours in animal models lacking enteral feedings [21]. Second, elevated inflammatory markers in circulation as early as the third postnatal day have been linked to an increased risk of neonatal morbidity [22].

### Maternal and Infant Data Collection

Maternal and infant data were abstracted from the electronic medical record. Maternal data included maternal age, gravida and para, ethnicity, multiple gestation, in-utero growth restriction, preeclampsia, premature rapture of membranes, use of antibiotics prior to delivery, pathology proven chorioamnionitis and mode of delivery. The diagnosis of preeclampsia was given by the obstetrical team based on high blood pressure and protein in the urine. Prolonged rupture of membranes was defined as membranes ruptured for >24 hours prior to delivery. Maternal antibiotics were defined as any antibiotics given to the mother within 24 hours of delivery for any reason besides surgical prophylaxis. Chorioamnionitis was defined by pathological examination of the placenta.

Infant data included gestational age, gender, birth weight, birth weight percentile < 10% (intrauterine growth restriction (IUGR)), weight at two weeks, APGAR scores at 1 and 5 minutes, SNAPPE II Score as a measure of severity of illness, number of sepsis evaluations, antibiotic treatments >5 days, postnatal day to initial feedings, number of days to full enteral feedings (defined as goal feedings of 150ml/kg/d), diet of initial feeding, diet at two weeks of age, volume of initial feeding and feeding advancement rates. Full parenteral and enteral nutritional profiles were obtained for each infant at two weeks of age. Gestational age and birth weight were also abstracted from the medical record for infants who did not satisfy inclusion criteria to assess for selection bias.

Chronic lung disease (CLD) was defined as requiring supplemental oxygen at 36 weeks’ corrected gestational age. Those infants who were transferred to an outside hospital near 36 weeks corrected gestational age who were still receiving supplemental oxygen were classified as having CLD. Retinopathy of prematurity (ROP) was defined as presence of any stage of retinopathy via an ophthalmologic examination by a qualified ophthalmologist. Intraventricular hemorrhage (IVH) and periventricular leukomalacia (PVL) were radiologically defined. Necrotizing enterocolitis (NEC) was defined as the presence of pneumatosis on abdominal radiographs.

### Fecal and Serum Sample Collection

The bedside nurse placed soiled diapers daily in a 4°C refrigerator. Study personnel collected the diapers and pooled fecal samples into 24hr aliquots, which were stored at -80°C until analysis. Discarded serum samples stored at 4°C were collected from the central laboratory within 48 hours of collection and stored at -80°C until analysis. Fecal and serum samples were included for analysis if they were collected at 2 weeks postnatal age +/- 4 days.

#### Fecal Lysates Preparation

Frozen fecal samples were thawed on ice for 30 minutes and then weighed. 100mg of sample was placed in a 2 mL Eppendorf tube and 1 mL of lysis buffer added. Lysis buffer consisted of 20% glycerol, 5mM EDTA, 0.2% CTAB, 0.05% Tween 20, and protease inhibitor. The samples were vortexed for 1–2 minutes until the sample was fully suspended. The samples were then homogenized in a tissue homogenizer and an additional 1 mL of lysis buffer was added. The sample was centrifuged at max speed (13,000x g) for 30 minutes and transferred to a new tube. Protein concentrations were determined using the BCA Protein Assay as described in manufacturer’s instructions (Pierce Protein Biology Products).

#### TLR ELISA Assay

Fecal lysates were prepared as described in the previous section and their protein concentrations were measured. 50 micrograms of protein were used per well for the ELISA assays. TLR4 and TLR9 ELISAs were purchased from MyBioSource and the assays were performed according to the manufacturer’s instructions. All samples were analyzed in duplicate. 50 **μ**L of standards or sample were added to the 96 well plate and 5 **μ**L of balance solution was added per well. 100 **μ**L of conjugate antibody was then added per well followed by incubation at 37°C for 1 hour. The plate was then washed 5 times with 250 **μ**L of 1x wash solution. This was followed by the addition of 50 **μ**L of Substrate A and 50 **μ**L of substrate B per well and incubation for 15 minutes. Finally 50 **μ**L of Stop Solution was added per well and the Optical Density at 450 nm was determined using a microplate reader. A standard curve was calculated using the standards provided by the manufacturer. The concentration of TLR4 and TLR9 proteins was determined based on the samples’ correspondence to the mean absorbance from the standard curve. The TLR4 and TLR9 values are expressed as ng/mL.

#### Fecal and Serum Cytokine Profiling

Fecal or serum samples were evaluated by a ten plex pro and anti-cytokine panel including: IL-10, IL-17A, IL1-RA, IL-6, IL-8, IP10, MIP1B, RANTES, TNFα, and VEGF. (Bio-Plex Precision Pro Human Cytokines, Bio-Rad, Hercules CA) Fecal and serum samples were also evaluated for acute phase reactants including: CRP, SAP, Haptoglobin and A2M using the same methodology (Bio-Plex Precision Pro, Human Acute Phase Assay Panel, Bio-Rad, Hercules CA). Multiplex Assays were performed using the Luminex Magpix machine, according to manufacturer’s instructions. Briefly, for the serum samples, serum was thawed on ice for 30min. 12.5 **μ**L of serum was then used per well for the cytokine assay, 5 **μ**L per well was used for the acute phase protein assays, 25 **μ**L was loaded for fecal samples. All samples were run in duplicates and the same standard curve was used for the entire experiment.

### Statistical Analyses

Continuous variables with normal distribution are expressed as mean ± standard deviation (SD) and non-parametric variables are expressed as the median with minimum maximum range or the interquartile range (IQR). Prevalence of variables and outcomes between groups are expressed as a percentage. Because the cytokine data were skewed, cytokine levels in fecal and serum samples as well as cytokine ratios were compared between the groups using the Kruskal-Wallis nonparametric statistical hypothesis test.

Linear and regression models were created through stepwise procedures to estimate the contribution of late initiation of enteral feedings on measure intestinal inflammation and neonatal morbidities. We conducted stepwise analysis of potential confounders in three sequential categories: (1) infant characteristics—gestational age, SNAPPE II, Apgar at 1 minute, Apgar at 5 minutes, growth restriction, and receipt of early antibiotics; (2) nutritional variables—diet at first feed, percent of feedings as breast milk, and total macronutrient delivery; and (3) neonatal morbidities—ROP, CLD, PVL, and the presence of two or more morbidities. If a variable was found to be significant, it was left in the model prior to adjusting for factors in the next category. The most parsimonious model is shown leaving in variables with a p value of 0.10 or lower or other variables shown to be important potential confounders in prior literature. Each cytokine measure was log transformed to satisfy normality assumptions of the parametric regression models. All analyses were performed using Stata version 13 (StataCorp, College Station, Texas).

## Results

Of 301 potential candidates, 27 infants were excluded for culture positive sepsis and 130 preterm infants met the inclusion criteria. Infants not included in the study were of greater gestational age (30.4±1.8 vs. 29.4±2.2 weeks) and higher birth weight (1555.4±392.1 vs. 1115.1±374.8 grams). Of the final study cohort, 79 infants received their first enteral feeding on or before the third postnatal day and were included in the Early group. 51 infants received their first enteral feeding after the third postnatal day and were included in the Late group.

### Baseline Maternal and Infant Characteristics

There were no significant differences in maternal characteristics between the two groups (Table 1). The infants in the Late group compared to the Early group were more likely to be born by Cesarean section (86 vs. 72%, respectively), however this difference was not statistically different (p = 0.06).

**Table 1:** Maternal and Infant Characteristics of Infants in the Early Feeding Group versus the Late Feeding Group.

Variables	Early (n = 79)	Late (n = 51)
**MATERNAL CHARACTERISTICS**		
Age (mean±SD)	31.0 ± 6.1	31.9 ± 5.9
Gravida (p50, range)	2 (1–11)	2 (1–10)
Para (p50, range)	0 (0–6)	0 (0–3)
Antenatal Steroids (%)	92	88
Antinatal Antibiotics (%)	39	32
PPROM (%)	19	10
Chorioamnionitis (%)	24	20
Preeclampsia (%)	20	26
Cesarean section (%)	72	86
Multiple (%)	48	36
Ethnicity (%)		
Caucasian	57	68
African American	13	12
Hispanic	9	8
Other	21	12
**INFANT CHARACTRISTICS**		
Gestational Age, weeks (mean, ±SD)	28.7±2.0	26.9±1.9**
Gender, Female (%), range	45.6	52.0
Birth Weight, g (mean, ±SD)	1242.2±367.9	907.5±293.0**
SNAPPE II Score (mean, ±SD)	13.6±12.5	29.7±18.4**
APGAR @1min (p50, range)	7 (1–9)	6 (1–8)*
APGAR @5min (p50, range)	8 (4–9)	8 (3–9)*
In utero growth restriction (%)	8.9	22.0*
Antibiotics exposure >48 hrs (%)	24.1	38.0*

Early = initial enteral feeding at the third postnatal day or less

Late = initial enteral feeding after the third postnatal day

* Early vs Late p<0.05;

** Early vs Late p<0.0001.

There were significant differences in the infant characteristics between the two groups. Infants in the Late group compared to infants in the Early group were on average almost two weeks younger at birth (26.9±1.9 vs. 28.7±2.0 weeks’ gestation) and thus were also of lower birth weight (907.5±293.0 vs. 1242.2±367.9 grams) (Table 1). In addition, infants in the Late group had higher severity of illness acuity scores (SNAPPE II, 29.7±18.4 vs 13.6±12.5) and were more likely to be growth restricted (22.0 vs. 8.9%). Finally, infants who received late enteral feedings had greater antibiotic exposure (38.0 vs. 24.1%) compared to those who were fed early before or on the third postnatal day. There were no sex differences between the two groups.

### Nutritional Practices

Infants in the Late group were more likely to receive breast milk as their first feeding (82.0 vs. 32.9%) (Table 2). The average time to the first feeding in the Late group was 7.3±2.6 days versus 2.1±0.7 days in the Early group. Although the initial feeding volumes of the two groups were not significantly different, the infants in the Late group were advanced more conservatively. Only 42.0% of the infants in the Late group were advanced faster than 10ml/kg/d compared to 69.6% in the Early group.

**Table 2:** Nutritional Milestones of Early versus Late Groups.

Variables	Early (n = 79)	Late (n = 51)
**Initial**		
Initial Feeding volume >10ml/kg/d (%)	16.5	6.0
BM @ First Feeding (%)	32.9	82.0**
Advancement Plan >10ml/kg/d (%)	69.6	42.0*
Days to 1st Feeding (days±SD)	2.1±0.7	7.3±2.6*
**2 weeks of Chronological Age**		
Breast Milk @ 2 weeks (%)	60.0	87.2*
Breast Milk/Enteral (%)	77.6	93.7**
Days to Full Feedings (days±SD)	13.1±5.5	22.7±16.8**
Weight (g±SD)	1308.7±372.3	962.1±262.8**
Weight above Birth Weight (%)	79.8	76.0
Daily Intake (±SD)		
Volume (ml/kg/d)	141. ±25.5	141.0±17.4
Calories (kcal/kg/d)	109.2±24.3	98.0±20.3*
Protein (g/kg/d)	3.2±1.2	2.6±1.4
Carbohydrate (g/kg/d)	11.3±2.3	12.1±1.6*
Fat (g/kg/d)	5.4±1.8	4.6±1.7*

* Early vs. Late p<0.05

** Early vs. Late p<0.0001.

Although the rates of breast milk consumption increased in both groups by two weeks of age, infants in the Late group continued to have higher rates of breast milk intake (87.2 vs. 60.0%). The daily total volume intake of parenteral and enteral nutrition combined was similar in the two groups. The infants in the Late group, however, received fewer calories (98.0±20.3 vs. 109.2±24.3kcal/kg/d) than the Early group. The macronutrient composition also differed between the two groups. Infants in the Early group received more fat (5.4±1 vs. 4.6±1.7g/kg/d); while, infants in the Late group received more carbohydrates (11.3±2.3 vs. 12.2±1.6g/kg/d).

Despite the observation that infants in the Late group started enteral nutrition later, were advanced more slowly, and took longer to achieve full feedings, the percent of infants who had regained weight above their birth weight by two weeks of age was similar in the Early and Late group (76.0 vs. 79.8%).

### Neonatal Mortality and Morbidities

Overall, the neonatal mortality of all infants in the study was 0.77% (n = 1; data not shown). The observed prevalence of neonatal morbidities (NEC, ROP, CLD, PVL, and intraventricular hemorrhage (IVH) were similar to nationally reported data (Table 3) [23], [24], [25], [26], [27].

**Table 3:** Univariate Analysis of Neonatal Morbidities by Group.

Outcomes (%)	Early (n = 79)	Late (n = 51)
NEC	6.3	10.0
ROP	16.7	52.1**
CLD	21.5	69.4**
PVL	0.0	6.0*
IVH	24.1	24.0
Comorbidities	8.0	25.0**

* Early vs. Late p<0.05;

** Early vs. Late p<0.0001

Necrotizing Enterocolitis (NEC); Retinopathy of Prematurity (ROP); Chronic Lung Disease (CLD); Periventricular Leukomalacia (PVL); Intraventricular Hemorrhage (IVH); Cormorbidities = The presence of 2 or more neonatal outcomes.

### Late enteral feedings is associated with an increase rate of neonatal morbidities

Contrary to the expected outcome, there were no differences in the prevalence of NEC between the two groups. However, consistent with our hypothesis that early nutritional practice impact rates of neonatal morbidities, infants in the Late group compared to those in the Early group were more than three times likely to have any stage of ROP (52.1 vs.16.7%), CLD (69.4 vs. 21.5%), and were more likely to have PVL (6 vs. 0%). In addition, the presence of two or more comorbidities was higher in the Late versus Early group (25.0 vs. 8%). There were no differences in the rates of IVH between the two groups. The fact that there were no differences in the rates of IVH between the two groups would suggest that these associations are not solely driven by gestational age and severity of illness. Indeed, this association persists even after adjustment for potential confounding variables between the two groups.

### Late enteral feedings is associated with increased neonatal morbidities after adjustment for potential confounders

The positive association of late enteral feedings with the neonatal morbidities of CLD, ROP and the presence of 2 or more morbidities remained significant after multivariate logistic modeling adjusting for potential confounders including maternal and infant characteristics, nutritional milestones, and other neonatal morbidities (Table 4). Compared to infants in the Early group, infants in the Late group had a significantly increased risk of developing CLD (OR 4.51, p = 0.002), ROP (OR 2.9, p = 0.03) or multiple morbidities (OR 3.42, p = 0.02).

**Table 4:** Adjusted Logistic Regression for the Odds of CLD and ROP with Late Initiation of Enteral Feedings.

Variables	OR (95% CI)	p-Value
**CLD**		
** Late Initial Feeding**	**4.51 (1.77–11.51)**	**0.002**
Gestational Age	0.90 (0.69–1.18)	0.45
SNAPPE II	1.03 (0.99–1.07)	0.20
Apgar 1 min	0.76 (0.57–1.02)	0.07
IUGR	4.08 (0.97–17.08)	0.05
**ROP**		
** Late Initial Feeding**	**2.90 (1.11–7.57)**	**0.03**
Gestational Age	0.57 (0.41–0.80)	0.001
SNAPPE II	0.99 (0.96–1.03)	0.68
IUGR	6.71 (1.70–26.43)	0.01
Total Fat	1.28 (0.97–1.70)	0.08
**Comorbidities**		
** Late Initial Feeding**	**3.42 (1.20–9.76)**	**0.02**
Gestational Age	0.55 (0.38–0.80)	0.002
SNAPPE II	1.02 (0.98–1.05)	0.33
IUGR	4.6 (1.08–19.64)	0.04

### Late enteral feedings is associated with an increase in select proinflammatory stool and serum cytokines

Compared to the Early group, fecal lysates from infants in the Late group had a 3-fold increase in the median expression of IL-8 (1.9±3.2 vs. 6. ±22.8 pg/ml), a proinflammatory cytokine. Concomitantly, there was a 3-fold reduction in the IL-1RA:IL-8 ratio (40±82.3 vs. 13.5±65.5) and a 2.5 fold reduction in the IL-10:IL-8 ratio in the same infants (5.3±6.9 vs 2.1±5.4) (Table 5). Both IL-1RA and IL-10 are anti-inflammatory cytokines. These results suggest a shift towards more pro-inflammatory and less anti-inflammatory cytokines in the stools of infants in the Late group.

**Table 5:** Univariate Analysis of Fecal Cytokine Expression in Association with Early vs. Late Groups.

Variables	Early (p50±IQR)	Late (p50±IQR)
TLR4 (ng/ml)	3.2±0.8	2.7±1.0
TLR9 (ng/ml)	0.1±0.2	0. ±0.3
IL-10 (pg/ml)	10.3±11.0	9.8±18.3
IL-17A (pg/ml)	13.6±30.3	15.3±26.4
IL-1RA (pg/ml)	78.2±162.1	161.3±135.0
IL-6 (pg/ml)	0.7±2.9	0.7±2.5
IL-8 (pg/ml)	1.9±3.2	6.1±22.8*
MIP1B (pg/ml)	3.0±8.6	0.3±10.6
RANTES (pg/ml)	0.0±8.2	0.2±7.5
TNFα (pg/ml)	1.1±7.8	2.1±5.7
VEGF (pg/ml)	823.0±1015.3	1121.1±1536.2
A2M (pg/ml)	7286.1±12180.5	6289.5±9209.9
CRP (pg/ml)	113.0±288.3	86.7±266.4
SAP (pg/ml)	75.8±1065.5	100.5±550.3
IL-1RA:IL-8	40.5±82.3	13.5±65.5*
IL-10:IL-8	8.2±1.2	5.9±1.6*

* = Early vs Late p<0.05.

Analysis of serum samples (Table 6) demonstrated that infants in the Late group had a two-fold increase in serum median CRP expression (948.8±4371.5 vs 447.7±1227.4 ng/ml, p = 0.02). In contrast, there was a fifty percent reduction in the serum IL-10:IL-8 ratio (0.1±0.2 vs. 0.2±0.3).

**Table 6:** Univariate Analysis of Serum Cytokine Expression (in pg/mL) in Association with Early vs. Late Group.

Variables	Early (p50±IQR)	Late (p50±IQR)
IL-10	16.6±12.8	13.4±15.9
IL-17A	16.6±21.9	21.9±20.5
IL-1RA	175.8±316.2	316.2±569.1*
IL-6	22.8±29.6	29.6±62.1
IL-8	83.0±118.6	118.6±115.2
IP10	245.2±165.3	165.3±138.4
MIP1B	276.6±241.2	241.2±192.1
RANTES	8090.8±9017.9	9017.9±18606.4
TNFα	10.5±10.5	10.5±8.3
VEGF	39.0±43.1	43.1±173.5
A2M	2530000±2620000	2620000±3675743
Haptoglobin	7738.4±4420.1	4420.1±132226.9
CRP	447.7±1227.4	948.8±4371.5*
SAP	23892.7±27694.5	27694.5±46946.7
IL-1RA:IL-8	1.6±2.4	2.4±4.0
IL-10:IL-8	0.2±0.3	0.1±0.2*

* = Early vs Late p<0.05.

### Impact of late enteral feedings on intestinal inflammation adjusted for potential confounders

The positive association of late initiation of enteral feedings on intestinal inflammation as measured by both fecal IL-8 levels and IL-10:IL-8 ratios remained significant after step-wise linear regression modeling adjusting for maternal and infant characteristics (including gestational age, birth weight, severity of illness), nutritional milestones (including amount and composition of parenteral nutrition and amount and type of enteral feedings), and common neonatal morbidities (Table 7). In addition, total fat intake and the diagnosis of CLD were predictors of IL-10:IL-8 ratios in fecal samples.

**Table 7:** Adjusted Linear Regression Coefficients of Fecal IL-8 Levels and IL-10:IL-8 Ratio with Late Initiation of Feedings and Associated Predictor Variables.

Variables	B-Coefficient (95%CI)	p-Value
**Fecal IL-8 Level**		
** Late Initial Feeding**	**2.91 (0.31–1.61)**	**0.005**
Gestational Age	^-^1.35 (^-^0.29–0.05)	0.18
SNAPPE II	^-^1.70 (^-^0.05–0.004)	0.09
APGAR 1 min	0.87(^-^0.11–0.28)	0.39
APGAR 5 min	^-^2.26 (^-^0.80–0.05)	0.03
Total Fat	^-^2.43 (^-^0.38–^-^0.04)	0.02
Amount of Breast Milk	0.54 (^-^0.64–1.12)	0.59
**Fecal IL-10:IL-8 Ratio**		
** Late Initial Feeding**	**-1.03 (-1.82–024)**	**0.01**
Gestational Age	0.12 (-.0.09–0.32)	0.26
SNAPPE II	0.02 (-0.009–0.04)	0.20
Total Fat	0.19 (0.006–0.37)	0.04
CLD	1.03 (0.21–1.86)	0.02
Comorbidities	-0.89 (-1.8–0.05)	0.06

Contrary to an association between fecal markers of inflammation and the initiation of feedings that persisted even after adjustment for confounders, the significant differences in serum CRP, IL-1RA, and IL-10:IL-8 ratio between the Early and Late groups were eliminated after adjustment for gestational age (data not shown).

### Late enteral feedings remain linked to increased risk of intestinal inflammation and comorbidities in a restricted cohort of non-IUGR, ELBW infants

To further reduce the contribution of potential confounding by gestational age and birth weight that may not have been fully adjusted for in the previous modeling we restricted the cohort to non-IUGR, extremely low birth weight (ELBW) infants (<1000g). Using the stepwise linear and logistic regression modeling procedures described previously, there continued to be a persistent significant association between late enteral feedings and intestinal inflammation and between late enteral feedings and neonatal outcomes (Table 8). Specifically, Late enteral feedings continued to show an independent association with increased fecal IL-8 levels and decreased IL-10:IL-8 ratios. Additionally, late enteral feedings remained a significant independent predictor of CLD.

**Table 8:** Adjusted Linear and Logistic Regression for Main Outcome Effects by Late Initial Feedings in a Restricted Cohort of Non-Growth Restricted, Extremely Low Birth Weight (n = 45).

Intestinal Inflammation	B-Coefficient (95%CI)	p-Value
** Fecal IL-8 Level**	**1.62 (0.47–2.77)**	**0.007**
** Fecal IL-10:IL-8 Ratio**	**-1.56 (-2.83–0.29)**	**0.02**
**Neonatal Morbidities**	**OR (95% CI)**	**p-Value**
** CLD**	**5.97 (1.30–27.4)**	**0.02**
ROP	1.97 (0.31–12.69)	0.48

## Discussion

In a cohort of 130 premature infants, infants with late enteral feedings, defined as the initial enteral feeding after the third postnatal day, were more likely to demonstrate increased markers of intestinal inflammation and were at increased risk of common neonatal morbidities, including CLD, ROP or having two or more comorbidities. We chose a panel of pro and anti-inflammatory cytokines that have been previously shown to be markers of inflammation and to be associated with neonatal morbidities. The fecal markers that were significantly different between the two cohorts included IL-8 and IL-10:IL-8 ratio. IL-8 was significantly elevated and IL-10:IL-8 ratio significantly reduced in the Late group. IL-8 has been shown previously to be an important inflammatory marker in the development of both CLD [14] and ROP [13].

Clinical differences were present between the Early and Late groups identifying infant characteristics that when present remain a barrier in establishing early feedings. These characteristics included being of an extremely low gestational age and extremely low birth weight, increased severity of illness as measured by the SNAPPE score, presence of antibiotics and intrauterine growth restriction. These potential barriers and associated clinical perceptions need to be addressed to change bedside practice in initiating early enteral feedings. Additionally, and of particular biological importance, is the finding of a significant relationship between late feedings and intestinal inflammation and neonatal morbidities even after adjustment for these potential confounders. Potential confounding was rigorously addressed in two ways. The first was with traditional step-wise modeling including infant, nutritional, and morbidity variables. The second was by repeating this modeling sequence but in a restricted cohort of non- IUGR, extremely low birth weight infants to further reduce the influence of gestational age, birth weight, and severity of illness. Although, complete adjustment of confounding may not be possible, these data support the hypothesis that early nutritional practices influence the presence of intestinal inflammation as well as the risk of neonatal morbidities. In addition, these findings support the need for clinical studies to specifically exam the *practice* of nutrition and how these parameters influence gut health and neonatal outcomes.

The potential for the gut to act as the “motor” of systemic illness is a well-accepted hypothesis in the adult literature [7]. The concept that inflammatory responses in the gut may also be an inherent mechanism of neonatal diseases remains plausible [10]. We were not able to directly link intestinal inflammation with systemic inflammation. The differences observed in the levels of CRP and IL-10:IL-8 ratio between the two groups were explained by the differences in the gestational ages between the two groups. It is possible that this may have been a result of too small of sample size. However, late enteral feedings were important for both intestinal inflammation and neonatal morbidities. These data are supported by prior studies demonstrating clustering of NEC with other systemic morbidities such as ROP, CLD, and impaired neurodevelopment [28]. Although the exact source of cytokines could not be determined, the amount of breast milk exposure (a possible source of intestinal cytokines) was not a predictor of fecal cytokine levels, suggesting that endogenous production is a more likely source. Additional studies are needed to specifically link diet, intestinal inflammatory markers and systemic inflammatory response.

In the multivariate modeling, adjustment for breast milk intake did not change the association between late enteral feedings and increased fecal IL-8 levels. The necessity for early administration of breast milk and its immunonutrients to maximize its beneficial effect is supported by a recent animal data showing that late administration of breast milk does not reduce the incidence of necrotizing enterocolitis [29]. In our cohort, if breast milk was provided as the first feed, the mean day to the initial feeding was 5.5 versus 2.5 if the first feeding was formula (data not shown). Unlike formula, which is readily available, breast milk production especially in mothers of premature infants can be delayed several days. We hypothesize that late initiation of enteral feeding may offset the potential immunologic benefits observed with a breast milk diet. This study suggests that the biological trade-offs to wait for mother’s own milk versus using formula or donor milk earlier may need to be evaluated further. Studies are needed to identify the preferred nutritional strategy evaluating the combined influence of diet and timing for optimal postnatal intestinal development.

A limitation of this study is the retrospective selection of infants from a pre-existing biorepository. The need to have concurrent fecal and serum samples to evaluate the study objectives selected for infants with lower gestational ages and birth weights. However, if the finding of an association between late feedings and intestinal inflammation were to be attributed to gestational age or severity of illness alone, this selection process would have biased the results to the null. Rather, after performing a sub-group analysis on the most immature infants and adjusting for identified confounders in multivariate modeling, the delayed initiation of enteral feedings remained a strong predictor of increased fecal IL-8 levels and decreased fecal IL-10:IL-8 ratios.

There are a number of important implications of these data. This is the first human study to show that timing of the first enteral feeding might be critical to intestinal inflammation and the development on neonatal diseases independent of gestational age and severity of illness. We postulate that one of the mechanism by which delayed introduction of enteral feedings influences intestinal inflammation is by altering colonization of the GI tract and thereby contributing to impaired immune ontogeny and inflammatory responses. Another contributory explanation is the concomitant reliance on parenteral nutrition until feedings are well established. In human and animal studies, the exposure to parenteral nutrition leads to a proinflammatory phenotype including enhanced intestinal inflammatory responses with the transition to enteral feedings [30], [31], [32].

Given that this study was not a randomized controlled trial, but rather a retrospective cohort study, we cannot show clear causality and can only define associations between events. Therefore, the proposed hypothesis needs to be tested further in larger randomized human trials and in animal studies to establish causation and define mechanisms by which nutritional practices impact intestinal development, immune ontogeny, microbial colonization, and risk of neonatal disease.
